# Harnessing sacrificial bond kinetics for hydrogel self-strengthening

**DOI:** 10.1016/j.xinn.2025.100988

**Published:** 2025-06-10

**Authors:** Xiaojuan Wang, Hongbin Liu, Meng Gao

**Affiliations:** 1 State Key Laboratory of Biobased Fiber Manufacturing Technology, Tianjin University of Science and Technology, Tianjin 300457, China

## Introduction

Biological tissues can self-renew and upgrade by recombining building blocks like amino acids from their environment. This reflects an open, dynamic growth process that allows adaptation to mechanical changes. Artificial materials, however, are usually based on closed, static systems.^1^ They cannot interact with the environment or grow structurally, making them progressive damage until failure in cyclic loading due to the absence of a structural reconstruction mechanism.

To meet practical requirements, it is crucial to endow artificial materials with self-strengthening capabilities akin to biological structures to withstand cyclic fatigue behaviors encountered in daily use. In recent years, researchers have created various partially or fully self-healing materials. The self-stiffening materials during mechanical use — by incorporating composites or liquid crystal elastomers — have also been developed. Additionally, using mechanochemical approaches to reshape polymers have been explored. Mechanochemistry uses physical forces (e.g., grinding, extrusion, and shearing) instead of traditional energy sources (heat, light, and electricity) to break and recombine polymer bonds, achieving structural reconstruction. This interdisciplinary field connects polymer chemistry, materials science, and mechanics, offering a foundation for designing force-responsive soft materials. However, reshaping bulk solid materials using these molecular mechanisms to enhance their mechanical properties remains a significant challenge.^2^

**Figure 1:**
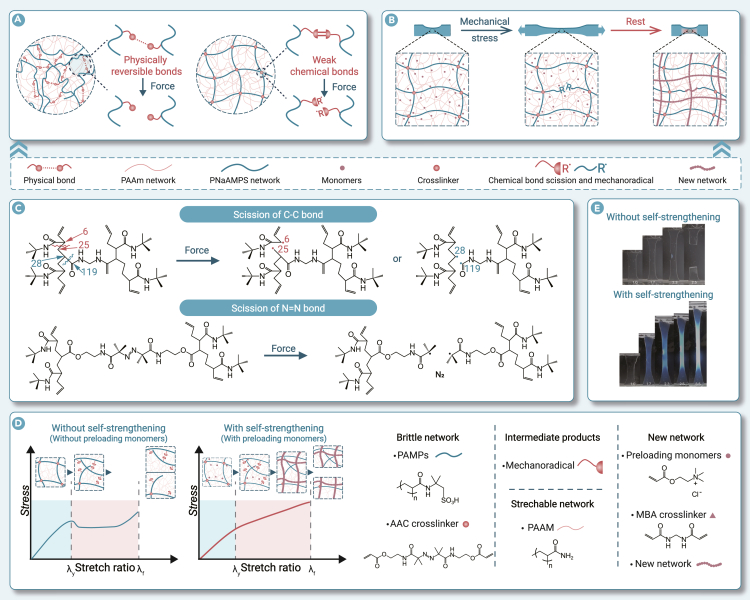
Scheme of harnessing sacrificial bonds for hydrogel self-strengthening (A) Schematic diagram of two types of sacrificial bonds. Reprinted (adapted) with permission.^1^ Copyright 2025 American Chemical Society. (B) Self-strengthening strategy of DN hydrogel materials. Reproduced with permission.^3^ Copyright 2025, The American Association for the Advancement of Science. (C) Mechanoradicals formed from the C–C bond rupture of PAMPS-MBA network (top) and the N=N bond rupture of PAMPS-AAC network (bottom). Reprinted (adapted) with permission.^1^ Copyright 2025 American Chemical Society. (D) Tensile stress-stretch curves and the corresponding molecular pictures of DN hydrogels without (left) and with (right) strengthening. (E) The crack strengthening via mechanoradical polymerization.^2^ Reproduced with permission.^2^ Copyright 2025, Springer Nature.

As a typical example, dual-network (DN) hydrogels, promising flexible materials, achieve enhanced toughness by incorporating an energy dissipation mechanism via sacrificial bonds. In the initial design, the two networks are constructed by short-chain and long-chain molecules crosslinked by covalent bonds, respectively. When stretched, the short-chain network breaks and dissipates energy but suffers permanent damage. After the first loading cycle, the gel cannot fully recover, and accumulated damage eventually leads to failure. To enable recovery, sacrificial covalent bonds are replaced with non-covalent (typically physical) bonds (Figure 1A). Under stress, the covalent-bonded network maintains structural integrity, while the physical network breaks bonds to dissipate energy. Under unloading, the physical network reforms, providing both enhanced toughness and self-healing capabilities.^4^

Furthermore, mechanochemically responsive weak chemical bonds capable of generating mechanoradicals can serve as a sacrificial network in DN hydrogels (Figure 1A).^1^ The semi-open nature of hydrogels enables molecular exchange through their interfaces, allowing DN gels immersed in monomer solutions to absorb monomer via diffusion.^1^^,^^5^ When a DN hydrogel involving a mechanically sensitive weak-bond network is deformed, the network rupture triggers generating numerous mechanoradicals, which subsequently initiate polymerization of the diffused monomers to promote the formation of a new network.This process remains effective under repeated loading cycles, approving dynamic mechanical self-strengthening of hydrogel and realizing the “growth” mechanism similar to biological load-bearing tissues.

The dynamic self-strengthening of DN hydrogels depends on the formation time of new network. Mechanochemical reinforcement is driven by chemical bond rupture, a fundamental process in polymer failure. If bond reformation lags significantly behind scission under typical loading conditions and loading rates, stress concentration accelerates crack propagation, causing premature failure instead of self-strengthening. Thus, controlling the timing of weak bond rupture and reformation—known as sacrificial bond kinetics—is essential. However, progress is limited by the high energy required for bond breaking, low mechanoradical production, and the lack of suitable monomers or crosslinkers. These issues prevent timely network regeneration. To overcome these challenges, it is important to develop weak sacrificial bonds with high energy dissipation efficiency and improved mechanoradical generation, and introduce more standby reconstruction monomers and crosslinkers for faster mechanochemical responses and rapid strengthening. This enhances the use of mechanochemistry in designing polymer mechanics.

## Regulation of sacrificial bond dynamics from scission to reformation

A research team from Hokkaido University, led by Professor Jianping Gong, has developed a DN hydrogel that self-strengthens upon mechanical deformation. The hydrogel utilizes N,N′-methylenebis(acrylamide) (MBA) as the co-crosslinking agent, poly(2-acrylamido-2-methylpropanesulfonic acid) sodium salt (PNaAMPS) as the brittle network, and poly(acrylamide) (PAAm) as the stretchable network. Upon mechanical deformation (e.g., stretching or compressing), weak C–C bonds in PNaAMPS or MBA break, collapsing the brittle network and generating mechanoradicals. These radicals, combined with the introduced additional monomers and crosslinkers, reconstruct the network, enabling the hydrogel to self-strengthen (Figures 1B and 1C).^3^ However, the C–C bond fracture consumes high energy and produces insufficient free radicals. To improve this, azoalkane crosslinker 2,2’-azobis-[2-methyl-N-(2-ethylpropenoate)]-propionamide (AAC) was synthesized for the brittle network. Its N=N bond cleavage, requires relatively low energy, generates five times more mechanoradicals than MBA (Figure 1C).^1^

Recently, the research team achieved real-time self-strengthening of “repair while damage” by introducing AAC weak sacrificial bonds.^2^ This approach enhances mechanoradical concentration, accelerating new network formation by 100 times.Specifically, the DN hydrogels consist of AAC-crosslinked poly(2-acrylamid-2-methyl-1-propanesulfonic acid (PAMPS) and MBA-crosslinked PAAm preloaded with monomers and crosslinkers to form a new network. Under mechanical forces (such as stretching or shear force), the weaker AAC covalent bonds break preferentially compared to C-C backbones and traditional MBA crosslinks, generating more mechanoradicals.These radicals increase active site density, promoting rapid polymerization of preloaded components and forming new networks (Figure 1D).^2^ The reaction kinetics depend on the concentrations of mechanical radicals, monomers, and crosslinkers within the hydrogel. Insufficient radicals or slow deformation delay strengthening. Results showed that this strategy reduces the network formation time to 20-50 s, whereas traditional DN hydrogels typically require minutes to hours. This acceleration allows prompt self-strengthening during deformation, inhibiting crack propagation and preventing premature failure. Compared to DN hydrogels without preloaded components, those with preloading exhibited a significantly reduced fracture stretch ratio and increased fracture stress, indicating considerable self-strengthening capacity (Figure 1E).^2^

## Concluding remarks and perspectives

By converting bond scission into a catalyst for network reconstruction, Gong et al. uncovered a self-strengthening mechanism that challenges classical fracture mechanics. The approach reveals the strengthening mechanism of force-induced weak bond breaking and reformation, achieving crack strengthening and prolonged durability. The mechanism is rate dependent and adjustable, applicable to other polymer materials, and complements existing strengthening strategies. This work will drive the on-demand design of functional materials with rate-dependent behavior via mechanochemistry. However, despite their exceptional performance, self-strengthening hydrogels face practical challenges that hinder their broader application.

Rate dependence paradox: Traditional polymers gain strength at higher loading rates due to limited chain relaxation. In contrast, self-strengthening hydrogels become stronger at lower rates, where mechanochemical reactions have more time to occur. This “slower-is-stronger” effect restricts their use in dynamic applications such as impact protection, which demand rapid response under high loading rates. Addressing this contradiction is key to expanding their applicability.

Fatigue resistance and long-term stability: Self-strengthening improves hydrogel durability, but repeated loading can still cause irreversible damage due to depleted free radicals or insufficient monomers, leading to failure. To enhance fatigue resistance, dynamic processes must be precisely controlled to balance bond formation and breakage. Moreover, sustainable “metabolic” mechanisms, such as self-replenishing networks inspired by biological tissue, should be developed.

Mechanics-function trade-offs: Current self-strengthening designs prioritize mechanical improvements, often sacrificing hydrogel functionality such as biocompatibility, electrical conductivity, or environmental responsiveness. Achieving a collaborative mechanics-function design is key to future applications.

As these challenges are addressed, self-strengthening hydrogels could significantly impact fields like flexible electronics and biomedicine, making materials “stronger with use.” As we advance toward self-strengthening hydrogels governed by reaction kinetics, the combination of molecular-scale bond engineering and macro-performance tuning promises to redefine the boundaries of soft-matter science.

## Funding and acknowledgments

The authors acknowledge support by the 10.13039/501100001809National Natural Science Foundation of China (52203049), the Tianjin Nature Science Foundation (23JCYBJC01020), the 10.13039/501100002858China Postdoctoral Science Foundation (2023M732613), and the Tianjin Youth Science and Technology Talent Foundation (no. QN20230337).

## Declaration of interests

The authors declare no competing interests.
